# The danger of non-exhaustive quality measures: requiring hip fracture repair surgery within 48 hours – a case study

**DOI:** 10.1186/s13584-017-0189-5

**Published:** 2017-11-28

**Authors:** Kobi Peleg, Michael Rozenfeld, Avi Israeli

**Affiliations:** 1 National Center for Trauma & Emergency Medicine Research, Gertner Institute for Epidemiology and Health Policy Research, 52621 Tel-Hashomer, Israel; 2 School of Public Health, Tel-Aviv University, Tel-Aviv, Israel; 3 Hebrew University, Hadassah School of Public Health, Jerusalem, Israel

**Keywords:** Quality measures, Quality indicators, Hip fracture, Earlier surgery, DRG, Mortality

## Abstract

Quality measures are widely used globally in order to measure clinical performance and organizational efficiency of the healthcare systems. However, in a race to achieve certain numerically defined goal, the more important purpose of any organizational step being aimed at improving clinical outcomes could be overshadowed.

The introduction of the requirement to perform most hip fracture surgeries in the first 48 h of hospitalization by the Israeli Ministry of Health (IMOH) provides an interesting example of the complexity of this phenomenon. In 2004, the IMOH decided that hospitals would receive the full DRG payment for hip fractures operations only in cases in which the operation is performed within 48 h of hospitalization. In 2013, the IMOH proceeded to designate the proportion of less than 48 h surgeries as an official quality parameter for comparing hospital performance.

Despite the widely acknowledged and proven clinical benefit of earlier surgery for hip fracture patients, the desired proportion of such surgeries in a given population is not easily defined for a given population, as a significant number of patients may be unsuited for immediate surgery due to medical instability, having a serious co-morbidity or receiving anticoagulant treatment. Rushing these patients to surgery can be therefore expected to have a negative effect on their outcomes, and the subsequent increase in hip fracture mortality recorded in Israel after 2013 may be a result of that.

This example suggests that designating an organizational quality measure without adjusting it for the patient’s medical condition may make it too inaccurate to guide healthcare policy.

Utilization of clearly delineated quality measures to assess and guide clinical practice is commonplace in most advanced healthcare systems all over the world. When uniformly defined for all hospitals in a specific country, quality measures may provide a valuable indicator of the system’s performance through benchmarking (comparison between different hospitals) and thus facilitate the patient’s choice of providers. By influencing the organizational parameters of clinical practice, they also have the potential to improve the level of treatment given to the patients, both in terms of user experience and better outcomes.

However, there are a several challenges that may arise when using quality measures to track system performance. These issues must be recognized and, if necessary, addressed in order to prevent a situation where performance measurement no longer drives positive change. First, the relevant population must be explicitly and accurately defined. Second, organizations must take care not to turn quality indicator achievements into goals in their own right, as the patient’s benefit should always remain the ultimate goal of the healthcare system and the standard it is measured by. Third, a valid measure of performance should not be treated as the sole measure of performance. It must be understood that sometimes there may be other dimensions not captured in this particular measure. These challenges tend to be contingent on one another – if the population is not clearly defined for a specific measure this may increase pressure to achieve 100% adherence, even if not clinically indicated. The adherence rate of this measure may then become the only lens through which this aspect in healthcare is viewed, especially if clinicians and other practitioners are late in providing feedback about the need to amend the measure.

With the ultimate goal of the healthcare system always being to provide optimal treatment to patients, achievement of organizational goals (including high scores on quality indicators) should be viewed as desirable only so long as it helps practitioners and organizations obtain better outcomes. Therefore, to be able to discuss actual improvement in any quality measure, the adherence rate should always be adjusted for relevant clinical outcomes and patient case-mix, and applied only to the relevant population, so that the goal of patients’ benefit is never lost in the labyrinth of organizational procedures.

A good example of such complexity in the application of quality measures is the measurement of the proportion of early hip fracture repair surgeries (performed within the first 48 h of hospitalization) with the goal of maximizing performance. This quality measure was introduced in Israel in 2013 by the Ministry of Health (MOH), for patients who are medically stable and do not have comorbid illness. The existence of this specific process measure is well-grounded in evidence: multiple studies from around the world, including Israel, confirm the significant impact of early hip fracture repair surgery on multiple outcomes, including length of stay in the hospital and mortality up to two years after hospitalization [1]. The requirement for hospitals to adhere to this guideline is also backed by a financial stimulus: since 2004 hospitals have received full reimbursement for hip fracture repair surgery only if it is performed within the first 48 h of hospitalization; if the operation is performed later – they receive only a proportion of the full rate.

However, from a clinical perspective, there is a certain controversy as to the desirability of maximizing the proportion of early hip fracture repair surgeries, as some patients may be unsuited for immediate surgery due to medical instability, having a serious co-morbidity or receiving anticoagulant treatment [2]. If these patients are rushed to surgery, they will not always benefit from the intended clinical effects; in fact they may be at a higher risk for adverse outcomes [3]. Clinically, these patients would likely benefit from delaying surgery until they are stabilized or taken off their medication regimen. However, as the organizational logic of following requirements to meet the established quality parameter pushes practitioners towards maximization of early surgeries (with the parameter being publicly compared between hospitals in the media), there is a danger of contraindicated patients being operated on anyway, thus compromising their survival. In a recent IJHPR article on the benefit of early hip fracture surgery in Israel, researchers noted a higher volume of co-morbidities among patients operated on after 2005, as compared to patients operated on before 2005 [4]; but an increase in mortality was recorded in some hospitals after 2013. This led the Ministry of Health to issue a recommendation to abstain from maximizing the achievement of this quality measure [5]. The current target defined by the Ministry of Health is for 85% of patients with hip fractures to undergo repair surgery within 48 h of the event. This target allows practitioners to use their judgment for each individual case. Selecting a target lower than 100% for this measure and other measures further assures that only the relevant population is measured, and can help overcome situations where the patient population may not have been as clearly defined. The situation could be further improved by in-depth characterization of the relevant population, for example, through developing a new measure of “medical stability”, which will include all the parameters that need to be taken into account for assigning patients to earlier surgery. In other words, if possible, to specify and define clearly the population included in (or excluded from) the measurement and only then expect achieving 100% adherence.

This example also highlights that reliance on a single parameter for assessing performance in a certain sphere creates a risk of this quality measure being not exhaustive enough, i.e. not including the whole spectrum of factors required for proper assessment. In order to minimize this risk, any quality measure being used should be adjusted for other considerations, e.g. patient’s health status, and specifically in our example: existence of serious co-morbidity or receiving anticoagulant treatment. If, after crossing a certain point in a key process parameter, deterioration in clinical outcomes is observed on population or organizational levels, the emphasis should change from maximization to optimization. Studies should be carried out that focus on recognizing these points of trend reversal, explaining the processes behind the change of trends, and characterizing the groups of patients that should be excluded from the general policy due to their specific epidemiological characteristics.

However, it is not enough to identify epidemiological factors in order to define exceptions and how to treat them; it is also important to understand the individuality of each patient. This is an important distinction between treatments and patients. Treatments can be classified into predefined, standardized categories, but patients will always remain individuals. A single quality measure will never cover all possible dilemmas that may emerge when an individual patient is compared to the standard on which the measure is based. The institutional structure of medical service delivery must be robust enough to simultaneously encourage an acceptably high standard of care and careful consideration of individual patient characteristics. Only then will patients benefit from truly optimal care.

## Conclusions

The example of requirement for maximizing the proportion of earlier hip surgeries in Israel clearly demonstrates the danger of utilizing non-exhaustive quality parameters. Medical policy makers should prefer organizational quality parameters conditioned by clinical outcome measures, while research should be focused on better understanding the interrelationships between all involved factors, in order to present the practitioners with better designed quality measures.
